# Data describing Upland cotton cultivars and advanced breeding lines used in Colombia

**DOI:** 10.1016/j.dib.2019.104140

**Published:** 2019-06-11

**Authors:** Oscar Burbano-Figueroa, Karen Sofia Montes-Mercado

**Affiliations:** The Plant Interactions Laboratory, Turipaná Research Center, Research Network for Industrial and Short-Season Crops, Corporación Colombiana de Investigación Agropecuaria (AGROSAVIA), Km 13 Via Monteria-Cerete, Cereté, 230558, Córdoba, Colombia

**Keywords:** Advanced breeding lines, Ramulosis, Upland cotton, LCER

## Abstract

In the last century, more than a hundred of cultivars were used in the cotton production system in Colombia. Breeding for cultivars adapted to tropical environments had been the main purpose of the Colombian agricultural research institutions dedicated to cotton. Data describing yield and fiber quality traits of these cultivars (and the introduced ones mainly from USA) is scattered across grey literature which reduces chances of discovering, accessing and assessing this information.This data article contains databases describing i) Colombian and introduced Upland cotton cultivars used in Colombia and ii) ramulosis-resistance scores of lines developed by the Colombian breeding program. The first database was constructed from data extracted from grey literature mainly produced by ICA and CORPOICA (rebranded today as AGROSAVIA), the Colombian agricultural research agencies. The second one describes the Cereté lines (LCER) database. These advanced breeding lines were developed for improved yield performance in tropical environments, specifically monsoon and savanna climates. The LCER dataset also describes the ramulosis field resistance of these cultivars. Ramulosis is an endemic disease in South America caused by *Colletotrichum gossypii* var. *cephalosporioides*. The data in this article supports and augments information presented in the research articles [1]: and [3].

**Table:** Specifications Table

Subject area	*Agricultural and Biological Sciences*
More specific subject area	*Crop Science and Plant Breeding*
Type of data	*Tables (Excel)*
How data was acquired	*Systematic search in technical reports (grey literature), books and papers using Google, Google Scholar, Agris and BAC.* *Experimental data from field plots used for constructing a resistance score of cotton advanced breeding lines again ramulosis*
Data format	*Raw*
Experimental factors	*Each cotton cultivar or advanced breeding line used in Colombia was searched for documents describing their use in Google, Google Scholar, Agris and BAC. BAC is the Colombian Agricultural Library and recently offers a repository for grey literature produced by ICA, CORPOICA and AGROSAVIA.* *.* *LCER data was obtained from technical reports available at AGROSAVIA and field experiments.* *Bibliographic information was recorded and organized using F1000Workspace.*
Experimental features	*Reported data for each cultivar was organized using several descriptors for breeding programs, designations, country origin, genealogy and bibliographic sources .*
Data source location	*Cereté, Córdoba, Colombia*
Data accessibility	*Data is with this article*
Related research article	*M. Moreno-Moran, O. Burbano-Figueroa, Field resistance of advanced breeding lines of upland cotton to ramulosis caused by Colletotrichum gossypii var. cephalosporioides, Crop Prot. 122 (2019) 49–56.*https://doi.org/10.1016/j.cropro.2019.04.008*.*[1]

**Table:** 

**Value of the data** • The datasets here provided consolidate information related with introduced and Colombian Upland cotton cultivars used in Colombia in the last century. • The data presented here describes the plant materials developed in the last half century by Colombian institutions providing a guidance paper for Colombian cotton breeders. • This document provides a reliable unified source of data for the cotton production system. Previously, data related with cultivars was dispersed across grey literature from several private and public institutions.This database is expected to provide the framework for collecting yield, fiber quality and other phenotypic traits of cotton cultivars described in these reports. • Data related with ramulosis resistance of advanced breeding lines is provided with the purpose of data comparison with other ramulosis resistance assays or its use in the design of new experiments.

## 1 Data

The datasets provided with this article describes i) Colombian and introduced Upland cotton plant materials used in Colombia and ii) ramulosis-resistance scores of breeding lines (BLs) developed by the Colombian breeding program in the last decade.

### 1.1 Plant material used in Colombia dataset

This file was constructed from data extracted from grey literature mainly produced by ICA and CORPOICA (rebranded today as AGROSAVIA), the Colombian agricultural research agencies. This dataset contains 220 entries that includes landraces, breeding lines, donor parents and commercial cultivars. Descriptors (Table 1) and references associated to the plant materials are included in this report.

**Table 1:** Summary of the descriptors used for detailing Upland cotton cultivars used in Colombia.

Descriptor	Descriptor Levels and commentaries
Plant Material Type	Breeding line, Cultivar, Donor parent, Landrace. Cultivar is used as synonymous of variety.
Cultivar Serie	This descriptor designates the germplasm collection or breeding program associated with a cultivar (STV, DP, LC, LCER)
Cultivar Code	A standardized code was assigned to each cultivar based on the ones used by the owner of the plant material. No blank spaces were included in this designation
Cultivar Trade Designation	Commercial branded name for cultivars
Country of Origin	Country were the plant material was developed. For the cultivars developed by CIRAD, it was not possible to establish where these materials were developed and consequently Africa was assigned as its origin.
Originator, Owner or Licenser	For those plant materials registered at Colombia, the name of the licenser was included in addition to the name of the originator or owner.
Parent 1	Description of parents for the Colombian plant materials
Parent 2	
Selection history	Identifies the plants selected at each generation of selfing since the original cross or individual selection
Selection method	Pedigree, Individual selection, Backcrossing. Individual selection stands for reselection of individuals for an specific cultivar
Transgenic	For those cultivars with transgenic genes: TRANSGENIC. Otherwise empty cells
Reference for pedigree or description 1	Technical report or paper where pedigree is described.
Reference for pedigree or description 2	
Market release (Year)	Release of the variety in the Colombian market.
Market exit (Year)	Year when the variety was not available anymore in the Colombian market
Adaptation region	Region for which the variety was intended to be released.
Additional description	Additional description that is not included in these descriptors.
ICA registration number	Registration number issued by ICA for plant variety protection.
ICA approval number	Registration number for cultivars approved by ICA to be used as varieties for specific adaptation regions. It is the equivalent of the PVP number in USA, but it is not widely available in public documents.

**Fig. 1:**
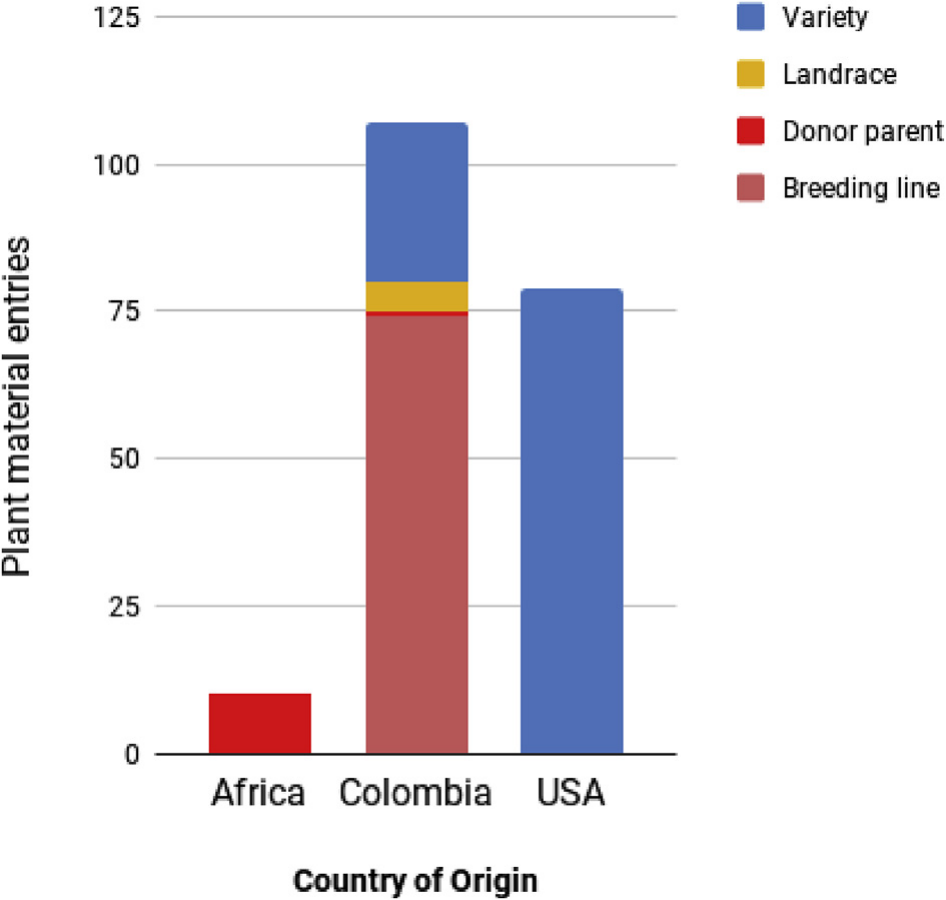
Distribution of cultivar entries reported in this paper according to country of origin and type of plant material. For the plant cultivars created in Africa by CIRAD was not possible to identify for all of them a specific country of origen and they are aggregated by continent.

The origin of the plant materials here described is mainly Colombian and American. It was not possible to verify the origin of 24 cultivars which contains some putative entries of Brazilian origin. Cultivars are mainly dominated by American introduced varieties while accessions of African origin (mainly from the CIRAD cotton program) were only used as donor parents in the Colombian breeding program (Fig. 1). More than two thirds of the Colombian entries are BLs (Fig. 1). Colombian BLs and cultivars belong to three collections (Cultivar serie):

• Gossica breeding lines (GS). This collection was developed by the Instituto Colombiano Agropecuario (ICA) in the 1960s–70s and released them as commercial cultivars during the 1970s–80s. Some of these lines were used for the LC cotton breeding program [1].
• LC breeding lines (Líneas Cesar). These lines were created by CORPOICA in the 1990s using accessions of African origin developed by CIRAD, GS lines, STV115 and LC8590. LC8590 is a Colombian accession without records about its origin. Seven commercial cultivars were released from this collection in the 2010s: Gaitana M109, Corpoica M123, Caribeña M129, Oro Blanco M151, Llanera M110, Vallenata M135, and Sinuana M137 [1].
• LCER breeding lines (Líneas Cereté). This collection was obtained from crosses between LC breeding lines and cultivars of American and Brazilian origin. CORPOICA released the cultivars LCER044 and LCER007 as commercial cultivars [1].

Recently, AGROSAVIA released in the Colombian market cultivars of the LC collection containing transgenic genes for glyphosate resistance (*cp4-epsps*) and Cry production *(cry1Ac* and *cry2Ab*) with the designations of Nevada 123 OMG (OMG stands for genetically modified organism), Oasis 129 OMG and San Juanera 151 OMG (ICA resolution numbers 32582, 32583 and 32584 respectively). These cultivars belonged to the collection LCBG2RF.

Besides the files describing these cultivars provided with this paper (Appendix A), a permanent link for this dataset was created for future updates including new cultivars and/or additional information for the reported cultivars (https://data.mendeley.com/datasets/4gj74rhv72/1).

### 1.2 Ramulosis resistance of LCER collection

The LCER advanced breeding lines were developed for improved yield performance in tropical environments, specifically monsoon and savanna climates. The LCER database describes the ramulosis field resistance of these cultivars. Ramulosis is an endemic disease in South America caused by *Colletotrichum gossypii* var. *cephalosporioides*. This dataset supports and augments information presented in the research article [3].

## 2 Experimental design, materials, and methods

### 2.1 Description of Upland cotton cultivars used in Colombia

Google, Google Scholar, Agris (http://agris.fao.org/agris-search/index.do) and BAC (The Colombian Agricultural Library) (https://repository.agrosavia.co/) databases were searched for the names of the cultivars belonged to the GS, LC and LCER series and their ancestors. The terms cotton and cultivars in Spanish (“algodón” and “variedades”) and “Colombia” were used for increasing the chances to identify additional information of any cultivar used in Colombia. Searches were complemented with previous reports that listed cultivars and the records for market release of cultivars issued by ICA, the regulating agricultural agency in Colombia.

### 2.2 Ramulosis resistance of LCER advanced breeding lines

Data related with ramulosis resistance was estimated from field experiments developed at two different locations (Turipaná and Motilonia RC) using ABLs and/or cultivars of the lines LCER and LC. Motilonia RC is located at the municipality of Codazzi (Cesar Valley, Cesar State, Northeastern Colombia) (coordinates 10.000, −73.250). The Cesar Valley is the second largest cotton producing region in the Caribbean and exhibits a dry savanna climate (As) according to the Koppen classification. Turipaná RC is located at the municipality of Cereté (Sinú Valley, Córdoba State, Northwestern Colombia) (coordinates: 8.850, −75.819). The Sinú Valley is the main cotton growing area in the Caribbean. The Sinú Valley exhibits a wet savanna climate (Aw).

Advanced breeding lines were arranged in triplicate plots in a RCBD design.This data is aggregated at plot (repetition) level showing the frequency for each disease level at the thirteen week after planting and different estimates of disease severity. Experiments are described in Ref. [3].

Ramulosis severity was estimated using a modification of a previously described scale [2] that allows better differentiation of plant resistance under high disease pressure conditions (Table 2). Both scales, the one presented here and the one described in Ref. [2], include 6 severity levels (p0 to p5), but they differ in the lowest severity level observed in the field. Under the high disease pressure observed in the experiments here described there was no presence of healthy plants (Level 0). At low disease pressure, no dwarf plants are observed (Level 6).

**Table 2:** Data describing the resistance of LCER ABLs to ramulosis.

Column Headers	Definition
LOC	Location of the experiment: MOT for Motilonia and TUR for Turipaná
WAP	Weeks after planting
ABL	Advanced Breeding Lines ID
p0^a^	Level 1. Plants with star-shaped lesion scattered in the lower third of the canopy
p1	Level 2. Numerous lesions all over the plant and curling with leaves surrounding meristems heavily infected
p2	Level 3. 1–3 sprouts
p3	Level 4. 4–10 sprouts
p4	Level 5. Plants exhibiting more than 10 sprouts
p5	Level 6. Dwarf plants with development of multiple vegetative branches
INC	Disease incidence
SPR(2–5)	Sprouting occurrence (frequency for severity levels from 2 to 5)
RAM(0–5)	Ramulosis Infection Intensity Index estimated as is described in Ref. [3]
RSC	Ramulosis Resistant Score
IDX	Disease Index (arithmetic estimation of severity)

a p denotes frequency for each disease severity level observed in the field. p0 corresponds to the lowest occurring severity level that for the experiment here described is Level 1. Healthy plants (Level 0) were not observed in this experiment.

## References

[1] Burbano-Figueroa O., Montes-Mercado K.S., Pastrana-Vargas I.J., Cadena-Torres J. (2018). Introducción y desarrollo de variedades de algodón Upland en el sistema productivo colombiano: una revisión. Rev Cien Agri.

[2] Moreno-Moran M., Burbano-Figueroa O. (2017). Dynamics of cotton ramulosis epidemics caused by Colletotrichum gossypii var. cephalosporioides in Colombia. Eur. J. Plant Pathol..

[3] Moreno-Moran M., Burbano-Figueroa O. (2019). Field resistance of advanced breeding lines of Upland cotton to ramulosis caused by Colletotrichum gossypii var. cephalosporioides. Crop Protect..

