# Supplementary material for: Data describing Upland cotton cultivars and advanced breeding lines used in Colombia
Source: Data Brief. 2019 Jun 11;25:104140. doi: 10.1016/j.dib.2019.104140 (PMC6595403; doi:10.1016/j.dib.2019.104140)
Supplement: Supplementary file 4 [file mmc4.doc]

# References describing cotton cultivars used in Colombia

#

[(Arévalo 2002)](http://f1000.com/work/citation?ids=5579294&pre=&suf=&sa=0)

[(Barragán Quijano et al. 2018a)](http://f1000.com/work/citation?ids=6369411&pre=&suf=&sa=0)

[(Barragán Quijano et al. 2018b)](http://f1000.com/work/citation?ids=6372612&pre=&suf=&sa=0)

[(Barragán Quijano et al. 2018c)](http://f1000.com/work/citation?ids=7001081&pre=&suf=&sa=0)

[(Brochero 1987)](http://f1000.com/work/citation?ids=4481794&pre=&suf=&sa=0)

[(Burbano-Figueroa et al. 2018)](http://f1000.com/work/citation?ids=6319208&pre=&suf=&sa=0)

[(Cadena Torres et al. 2001)](http://f1000.com/work/citation?ids=3858617&pre=&suf=&sa=0)

[(Cadena Torres et al. 2003)](http://f1000.com/work/citation?ids=5588888&pre=&suf=&sa=0)

[(Corpoica 1999)](http://f1000.com/work/citation?ids=6048670&pre=&suf=&sa=0)

[(CORPOICA 2017)](http://f1000.com/work/citation?ids=3858603&pre=&suf=&sa=0)

[(Diaz Delgado 2002)](http://f1000.com/work/citation?ids=5589195&pre=&suf=&sa=0)

[(Espitia Camacho 1991)](http://f1000.com/work/citation?ids=6361488&pre=&suf=&sa=0)

[(Espitia Camacho et al. 2008)](http://f1000.com/work/citation?ids=5576893&pre=&suf=&sa=0)

[(Jarma Orozco et al. 2002)](http://f1000.com/work/citation?ids=5581466&pre=&suf=&sa=0)

[(Mendoza Olivella 2000)](http://f1000.com/work/citation?ids=6211213&pre=&suf=&sa=0)

[(Mendoza Olivella 2002)](http://f1000.com/work/citation?ids=5579799&pre=&suf=&sa=0)

[(Mendoza Olivella et al. 1979)](http://f1000.com/work/citation?ids=5580525&pre=&suf=&sa=0)

[(Mendoza Olivella et al. 2000)](http://f1000.com/work/citation?ids=5569274&pre=&suf=&sa=0)

[(Mendoza Olivella et al. 2003)](http://f1000.com/work/citation?ids=5573162&pre=&suf=&sa=0)

[(Mercado de Duque et al. 1998)](http://f1000.com/work/citation?ids=4707308&pre=&suf=&sa=0)

[(Peñaloza 2002)](http://f1000.com/work/citation?ids=4297857&pre=&suf=&sa=0)

[(Rache Cardenal 2011)](http://f1000.com/work/citation?ids=3873905&pre=&suf=&sa=0)

[(Sierra et al. 2010)](http://f1000.com/work/citation?ids=5246730&pre=&suf=&sa=0)

[(Urrea Gómez and Agamez 2001)](http://f1000.com/work/citation?ids=6210571&pre=&suf=&sa=0)

[(Urrea Gómez and Garcés Gutierrez 2000)](http://f1000.com/work/citation?ids=6210581&pre=&suf=&sa=0)

[(Urrea Gómez and Mendoza Olivella 2002)](http://f1000.com/work/citation?ids=6210580&pre=&suf=&sa=0)

[(Urrea Gómez et al. 1999)](http://f1000.com/work/citation?ids=6210579&pre=&suf=&sa=0)

[(Urrea Gómez et al. 2000)](http://f1000.com/work/citation?ids=3858631&pre=&suf=&sa=0)

[(Vallejo de Cardona 1997)](http://f1000.com/work/citation?ids=5580523&pre=&suf=&sa=0)

[(Vallejo Rincón 1974)](http://f1000.com/work/citation?ids=6340733&pre=&suf=&sa=0)

[(Vallejo Rincón 1985)](http://f1000.com/work/citation?ids=5579045&pre=&suf=&sa=0)

#

[Bibliography](http://f1000.com/work/bibliography)

[Arévalo, M. ed. 2002. *Nuevas variedades y tecnologías para el manejo de suelos y malezas del algodonero en Colombia*. Bogotá.](http://f1000.com/work/bibliography/5579294)

[Barragán Quijano, E., Núñez Zarantes, V.M., Pastrana Vargas, I.J. and Cadena Torres, J. 2018a. *Nevada- 123 OMG: Variedad de algodón nacional con doble transgénesis*. AGROSAVIA.](http://f1000.com/work/bibliography/6369411)

[Barragán Quijano, E., Núñez Zarantes, V.M., Pastrana Vargas, I.J. and Cadena Torres, J. 2018b. *Oasis- 129 OMG: Variedad de algodón con doble transgénesis*. AGROSAVIA.](http://f1000.com/work/bibliography/6372612)

[Barragán Quijano, E., Núñez Zarantes, V.M., Pastrana Vargas, I.J. and Cadena Torres, J. 2018c. *San Juanera 151 OMG: Variedad de algodón para el Caribe Seco con doble transgénesis*. AGROSAVIA.](http://f1000.com/work/bibliography/7001081)

[Brochero, M.F. 1987. Importancia Socioeconómica del Cultivo del Algodón en Colombia. In: *La Producción de Semillas de Algodón para Siembra en Colombia*. Manual de Asistencia Técnica no. 41. Bogotá: Instituto colombiano Agropecuario (ICA), pp. 1–10.](http://f1000.com/work/bibliography/4481794)

[Burbano-Figueroa, O., Montes-Mercado, K.S., Pastrana-Vargas, I.J. and Cadena-Torres, J. 2018. Introducción y desarrollo de variedades de algodón Upland en el sistema productivo colombiano: Una revisión. *CIENCIA Y AGRICULTURA* 15(1), pp. 29–44.](http://f1000.com/work/bibliography/6319208)

[Cadena Torres, J., Mendoza Olivella, A., Torres A, G., Caicedo G., S., Bernal R, J. and Navas R, G. 2003. *Llanera M-110 variedad mejorada de algodón de fibra media para los Llanos Orientales*. 1era ed. Villavicencio: Corpoica.](http://f1000.com/work/bibliography/5588888)

[Cadena Torres, J., Urrea Gómez, R., Mendoza Olivella, A., et al. 2001. *Corpoica M-123 : nueva variedad de algodón de fibra media con adaptación al Caribe Húmedo*. Corpoica.](http://f1000.com/work/bibliography/3858617)

[Corpoica 1999. *Plan nacional para mejorar la competitividad y sostenibilidad del sistema de producción de algodón en Colombia*. Corpoica.](http://f1000.com/work/bibliography/6048670)

[CORPOICA 2017. *Variedad de algodón Sinuana M-137, adaptada a las condiciones de la subregión del Caribe Húmedo. Valle del Sinú, las Sabanas de Sucre y Bolívar, y el Bajo Magdalena*. Bogotá: CORPOICA.](http://f1000.com/work/bibliography/3858603)

[Diaz Delgado, A. 2002. Periodo y densidad de siembra recomendados para la variedad de algodón Gaitana M-109 en el Valle Cálido del Alto Magdalena. In: Corpoica ed. *El hombre y la nueva tecnología del cultivo del algodonero en Colombia*. Bogotá, pp. 136–141.](http://f1000.com/work/bibliography/5589195)

[Espitia Camacho, M.M. 1991. *Estabilidad fenotípica del rendimiento y calidad de la fibra en líneas promisorias de algodón (Gossypium hirsutum. L)*. Santafé de Bogotá (Colombia): Universidad Nacional de Colombia. Santafé de Bogotá.](http://f1000.com/work/bibliography/6361488)

[Espitia Camacho, M.M., Araméndiz Tatis, H. and Cadena Torres, J. 2008. Correlaciones y análisis de sendero en algodón *(Gossypium hirsutum    L.)* en el Caribe colombiano. *Revista Facultad Nacional de Agronomía, Medellín*.](http://f1000.com/work/bibliography/5576893)

[Jarma Orozco, A., Contreras C, A., Morales V, W. and Romero, J.L. 2002. *Surcos ultra angostos en la nueva variedad de algodón Corpoica M-123 en el Valle del Sinú.*. Bogotá: CORPOICA.](http://f1000.com/work/bibliography/5581466)

[Mendoza Olivella, A. 2002. Antecedentes y proyecciones del desarrollo de variedades de algodón adaptadas a los sistemas productivos de Colombia. In: Corpoica ed. *Plan Nacional para mejorar la Competitividad y Sostenibilidad del Sistema de Producción de Algodón en Colombia*. Bogotá.](http://f1000.com/work/bibliography/5579799)

[Mendoza Olivella, A. 2000. *Características de las nuevas variedades de algodón para el Caribe Seco (Valledupar, Bosconia y San Pedro).* Montería: Corpoica.](http://f1000.com/work/bibliography/6211213)

[Mendoza Olivella, A., Cadenas Torres, J., Barragán Quijano, E., et al. 2003. *Gaitana M 109 variedad rendidora de excelente calidad textil apta para las zonas secas del alto Magdalena*. Corpoica ed. Ibagué.](http://f1000.com/work/bibliography/5573162)

[Mendoza Olivella, A., Gómez Duque, J. and Vallejo Rincón, R. 1979. *Conozca las variedades colombiana de algodón: ICA Bravo, Gossica N 21, Gossica N 22, Gossica P 21.* Bogotá.](http://f1000.com/work/bibliography/5580525)

[Mendoza Olivella, A., Jarma Orozco, A., Castro Ortega, L. and Bornacelly Lopez, C. 2000. Caribeña M-129: variedad mejorada de algodón de fibra media para el Caribe Seco. *Plegable Divulgativo - Corporación Colombiana de Investigación Agropecuaria (Colombia)*.](http://f1000.com/work/bibliography/5569274)

[Mercado de Duque, M., Ramírez González, N. and Rodríguez Quijano, P. eds. 1998. Algodón. In: *Principales avances en investigación y desarrollo tecnológico por sistemas de producción agrícola*. Santafé de Bogotá, pp. 11–24.](http://f1000.com/work/bibliography/4707308)

[Peñaloza, E. 2002. Evaluación de los glifosatos usados como quema química y en post-emergencia en el cultivo del algodón. In: Bornacelly Lopez, C. and Arévalo, M. eds. *Estrategias de Organización, Comercialización Y Tecnológicas Para Mejorar la competitividad del sistema de producción del algodón en el Cesar y Guajira*. Memorias Foro Tecnológico. Bogotá: Plan Nacional de Algodón.](http://f1000.com/work/bibliography/4297857)

[Rache Cardenal, L.Y. 2011. Monitoreo del flujo de genes de algodón transgénico en la Agremiación Remolino sa (Espinal–Tolima). Master thesis. Universidad Nacional de Colombia.](http://f1000.com/work/bibliography/3873905)

[Sierra, C.M., Galvis, L., Trebilcok, A. and Cadena Torres, J. 2010. Comportamiento de la variedad NuOPAL (*Gossypium hirsutum* L.) bajo diferentes arreglos espaciales. *Temas Agrarios* 15(2), p. 66.](http://f1000.com/work/bibliography/5246730)

[Urrea Gómez, R. and Agamez, A. 2001. *Comportamiento agronómico de variedades comerciales de algodón en las sabanas de San Pedro- Sucre*. Montería: Plan Nacional de Algodón, Corpoica.](http://f1000.com/work/bibliography/6210571)

[Urrea Gómez, R. and Garcés Gutierrez, R. 2000. *Sinuana M-123: Características agronómicas y calidad de fibra. Próxima variedad de algodón para el Valle del Sinú*. Montería: Corpoica.](http://f1000.com/work/bibliography/6210581)

[Urrea Gómez, R., Garces Gutierrez, R., Aramendiz Tatis, H. and Mendoza Olivella, A. 2000. Nuevas variedades de algodón adaptadas al Valle del Sinú. In: CORPOICA and CONALGODON eds. *Aportes Tecnológicos a la Producción Competitiva Y Sostenible Del Algodonero en la Región Caribe*. Corpoica, pp. 7–16.](http://f1000.com/work/bibliography/3858631)

[Urrea Gómez, R., Garcés Gutierrez, R., Aramendiz Tatis, H. and Mendoza Olivella, A. 1999. *Nuevas variedades de algodón adaptadas al Valle del Sinú.* CORPOICA.](http://f1000.com/work/bibliography/6210579)

[Urrea Gómez, R. and Mendoza Olivella, A. 2002. *Características agronómicas de la variedad de algodón GMC 23-828 de adaptación específica a las Sabanas de San Pedro en Sucre.* Bogotá: Corpoica.](http://f1000.com/work/bibliography/6210580)

[Vallejo de Cardona, G. 1997. *Evaluación de caracteres agronómicos en cuarenta y ocho variedades de algodón.* Bogotá.](http://f1000.com/work/bibliography/5580523)

[Vallejo Rincón, R. 1974. Resistencia varietal del algodonero para el control de plagas. *Biblioteca Digital Agropecuaria De Colombia, Biblioteca Digital Agropecuaria de Colombia.*](http://f1000.com/work/bibliography/6340733)

[Vallejo Rincón, R. 1985. *Variedades de algodón en Colombia*. Bogotá: Instituto Colombiano Agropecuario (ICA).](http://f1000.com/work/bibliography/5579045)
